# Gene Expression and Fatty Acid Composition in Sea Buckthorn Seeds and Pulp During Fruit Development of Different Varieties

**DOI:** 10.3390/ijms262110396

**Published:** 2025-10-26

**Authors:** Nataliya V. Melnikova, Alexander A. Arkhipov, Yury A. Zubarev, Roman O. Novakovskiy, Anastasia A. Turba, Arthur G. Yablokov, Gleb N. Vladimirov, Sergey V. Osipenko, Anton A. Bashilov, Yury I. Kostyukevich, Eugene N. Nikolaev, Elizaveta A. Sigova, Ekaterina M. Dvorianinova, Daiana A. Krupskaya, Nikolai M. Barsukov, George S. Krasnov, Chengjiang Ruan, Elena N. Pushkova, Alexey A. Dmitriev

**Affiliations:** 1 Engelhardt Institute of Molecular Biology, Russian Academy of Sciences, 119991 Moscow, Russia; mnv-4529264@yandex.ru (N.V.M.); arkhipov.aleksandr2.0@gmail.com (A.A.A.); 0legovich46@mail.ru (R.O.N.); anastas.turba@gmail.com (A.A.T.); melarsoprol@mail.ru (A.G.Y.); sigova1567@gmail.com (E.A.S.); eugenevarenyuk@gmail.com (E.M.D.); zhernova.d@ya.ru (D.A.K.); keepter@yandex.ru (N.M.B.); gskrasnov@mail.ru (G.S.K.); pushkova18@gmail.com (E.N.P.); 2 Federal Altai Scientific Center of Agrobiotechnologies, 656910 Barnaul, Russia; niilisavenko@yandex.ru; 3 Project Center of Omics Technologies and Advanced Mass Spectrometry, 121205 Moscow, Russia; gleb.vladimirov@gmail.com (G.N.V.); ossipenko9191@gmail.com (S.V.O.); anton_bashilov@mail.ru (A.A.B.); yura542@gmail.com (Y.I.K.); ennikolaev@gmail.com (E.N.N.); 4 Institute of Plant Resources, Key Laboratory of Biotechnology and Bioresources Utilization, Ministry of Education, Dalian Minzu University, Dalian 116600, China; ruan@dlnu.edu.cn

**Keywords:** sea buckthorn, *Hippophae rhamnoides*, fruit development, fatty acid composition, gene expression, *KAS*, *FAT*, *SAD*, *FAD*

## Abstract

Sea buckthorn (*Hippophae rhamnoides* L.) is an oil crop with health benefits. Its fruits are rich in unsaturated fatty acids (FAs); however, the FA composition of the seeds and pulp differs significantly. To evaluate the expression levels of gene families that play a major role in FA biosynthesis, the transcriptomes of seeds and pulp at four fruit development stages were sequenced for five sea buckthorn varieties with diverse characteristics: Elizaveta, Inya, KP-686, Panteleevskaya, and Triumf. The results revealed that *FAD3 (07426)* and *FAD3 (05528)* are likely key genes for linolenic acid synthesis in seeds, while *FAD2 (21624)* is likely the main contributor to linoleic acid synthesis in both seeds and pulp. *SAD (18830)* primarily contributes to oleic acid synthesis in seeds, while *SAD (18830)* and *SAD (26748)* contribute to its synthesis in pulp. *FATA (14745)* and *FATA (14109)* are also implicated in FA synthesis in sea buckthorn fruits. Changes in the content of the main FAs in seeds and pulp correlated with the expression levels of the corresponding genes. KP-686 and Triumf differed the most from other varieties. These results are important for analyzing tissue-specific gene expression in seeds and pulp of sea buckthorn fruits, and they are promising for developing sea buckthorn varieties with improved oil composition.

## 1. Introduction

Sea buckthorn (*Hippophae rhamnoides* L.) is a tree crop whose fruits are rich in unsaturated fatty acids (FAs). The seeds contain high levels of linolenic acid (18:3, omega-3) and linoleic acid (18:2, omega-6), while the pulp contains a high level of rare palmitoleic acid (16:1, omega-7) [1,2,3]. However, the low oil content in the seeds and the high palmitic acid content in the pulp hinder the development and expansion of healthy sea buckthorn products. In order to improve these unfavorable characteristics and develop varieties with an optimal FA composition in the pulp, it is necessary to understand the mechanisms responsible for the differences in FA content between the pulp and the seeds.

Despite its value to the food and pharmaceutical industries [3,4,5,6,7,8,9], sea buckthorn has been relatively understudied from a genetic point of view [10]. Recently, however, high-quality genome assemblies of *H. rhamnoides* were obtained [8,11,12]. These assemblies are essential tools for molecular genetic studies of this useful plant, including the analysis of particular gene families.

Researchers have focused their attention on the genes involved in FA synthesis in plants. The following proteins take part in FA synthesis in plants, including sea buckthorn: beta-ketoacyl-acyl carrier protein (ACP) synthase II (KAS II), which is involved in the elongation of 16:0-ACP to 18:0-ACP; oleoyl-acyl carrier protein thioesterase (FATA) and palmitoyl-acyl carrier protein thioesterase (FATB), which play a role in terminating the synthesis of the growing FA chain and controlling the release of FAs; stearoyl-ACP desaturase (SAD), which converts stearic acid (18:0) to oleic acid (18:1); FA desaturase 2 (FAD2) and FA desaturase 6 (FAD6), which transforms oleic acid (18:1) to linoleic acid (18:2); FA desaturase 3 (FAD3) and FA desaturase 7/8 (FAD7/8), which convert linoleic acid (18:2) to linolenic acid (18:3) [1,11,13,14]. In our previous work, we identified the following genes in the sea buckthorn genome: four *KAS II*, six *FATB*, two *FATA*, nine *SAD*, three *FAD2*, five *FAD3*, one *FAD6*, and three *FAD7/8*. We also evaluated their expression levels in the variety Yantarnaya yagoda [15]. Previous studies also analyzed the expression of sea buckthorn genes that can be involved in oil synthesis [1,11,14]. These studies provided insight into the molecular mechanisms that form FA composition in sea buckthorn fruits. However, different varieties of sea buckthorn can significantly differ in their characteristics, including the FA composition of oil in their fruit pulp and seeds [16,17,18]. Previous studies predominantly focused on single sea buckthorn genotypes. Studying representative sets of different sea buckthorn varieties can help identify the key genes responsible for FA synthesis and explain the differences in FA composition between seeds and pulp and among different genotypes. This information could be used for breeding and genome editing to create improved sea buckthorn varieties. The present study aimed to analyze the expression of genes related to FA synthesis in seeds and pulp of five sea buckthorn varieties with different fruit characteristics, as well as to obtain data on their FA composition during fruit development.

## 2. Results and Discussion

### 2.1. Gene Expression Analysis

We performed transcriptome sequencing of seeds and pulp at four stages of fruit ripening for five sea buckthorn varieties (Elizaveta, Inya, KP-686, Panteleevskaya, and Triumf) on the Illumina platform. On average, approximately four million reads were generated for each sequenced cDNA library. The sequencing data were analyzed to obtain information on gene expression in the studied samples.

Further, a gene expression analysis was performed for the *KAS*, *FAT*, *SAD*, and *FAD* gene families, particularly for *KAS II (03443)*, *KAS II (10812)*, *KAS II (15320)*, *KAS II (26485)*, *FATA (14109)*, *FATA (14745)*, *FATB (03360)*, *FATB (07959)*, *FATB (17924)*, *FATB (18201)*, *FATB (24276)*, *FATB (28610)*, *SAD (07832)*, *SAD (18766)*, *SAD (18803)*, *SAD (18830)*, *SAD (18832)*, *SAD (21192)*, *SAD (23875)*, *SAD (26748)*, *SAD (28095)*, *FAD2 (12459)*, *FAD2 (21624)*, *FAD2 (27005)*, *FAD3 (05528)*, *FAD3 (07426)*, *FAD3 (10700)*, *FAD3 (24146)*, *FAD3 (24879)*, *FAD6 (25786)*, *FAD7/8 (02511)*, *FAD7/8 (07747)*, and *FAD7/8 (17598)* genes, which were identified in our previous study [15].

In general, these genes exhibited similar expression profiles during fruit development for all the studied varieties. However, the expression levels and profiles of genes within the same family differed greatly.

### 2.2. KAS II Expression

The expression levels of all *KAS II* genes (*KAS II (03443)*, *KAS II (10812)*, *KAS II (15320)*, and *KAS II (26485)*) were low. *KAS II (03443)* and *KAS II (10812)* exhibited the highest expression levels, peaking in seeds during the second stage of fruit development and also demonstrated significant change in expression during ripening (Table S1).

### 2.3. FAT Expression

**Figure 1:**
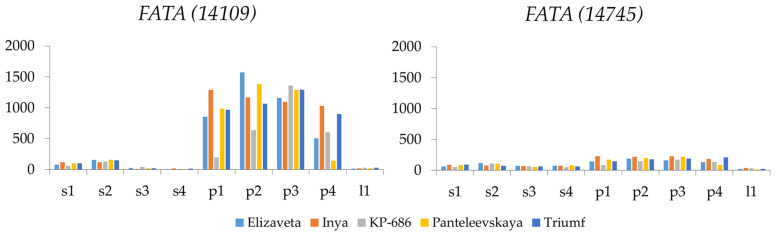
Expression of *FATA (14109)* and *FATA (14745)* genes in seeds (s) and pulp (p) at four fruit ripening stages (1, 2, 3, 4) and in leaves (l) of five *Hippophae rhamnoides* varieties: Elizaveta, Inya, KP-686, Panteleevskaya, and Triumf. CPM (counts per million) values are shown on the y-axis.

Among the *FAT* family, the *FATA (14109)* gene exhibited the highest expression level, which was tenfold higher in pulp than in seeds (Figure 1 and Table S1). Significant changes in the expression level of this gene were observed during fruit ripening in both seeds and pulp. However, expression in pulp largely depended on genotype. *FATA (14109)* was barely expressed in leaves. *FATA (14745)* was significantly expressed in pulp (Figure 1 and Table S1), and its expression changes during fruit development correlated with expression changes of *FATA (14109)*. The expression levels of *FATB (03360)*, *FATB (07959)*, *FATB (17924*), *FATB (18201)*, *FATB (24276)*, and *FATB (28610)* were relatively low (Table S1). Therefore, *FATA (14745)* and, in particular, *FATA (14109)*, are likely the primary contributors to FA synthesis in sea buckthorn pulp among the *FAT* genes.

### 2.4. SAD Expression

**Figure 2:**
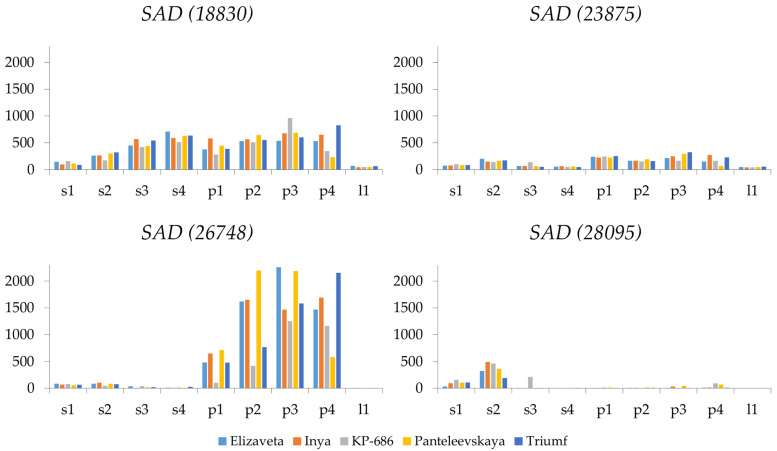
Expression of *SAD (18830)*, *SAD (23875)*, *SAD (26748)*, and *SAD (28095)* genes in seeds (s) and pulp (p) at four fruit ripening stages (1, 2, 3, 4) and in leaves (l) of five *Hippophae rhamnoides* varieties: Elizaveta, Inya, KP-686, Panteleevskaya, and Triumf. CPM (counts per million) values are shown on the y-axis.

Among the *SAD* genes, *SAD (18830)* exhibited a high expression level in seeds and pulp (Figure 2 and Table S1). The maximum expression level of *SAD (18830)* occurred at the third and fourth stages of fruit development. Meanwhile, *SAD (26748)* exhibited the highest expression level in pulp and was barely expressed in seeds. The expression profile of *SAD (26748)* in pulp differed somewhat from that of *SAD (18830)*, although *SAD (26748)* was also more highly expressed at later stages of fruit development. *SAD (26748)* and *SAD (18830)* were expressed at low levels in sea buckthorn leaves, confirming their role in FA synthesis specifically in fruits. *SAD (23875)* had an increased expression at all stages of fruit ripening in pulp and at the second stage in seeds, while the level of *SAD (28095)* was higher in seeds at the second stage (Figure 2 and Table S1). As for the other *SAD* genes, *SAD (18803)*, *SAD (18832)*, and *SAD (21192)* were barely expressed, while the expression levels of *SAD (18766)* and *SAD (07832)* were relatively low and did not vary significantly between organs or development stages. However, expression of *SAD (07832)* was increased in pulp and at the later ripening stages in seeds (Table S1). Since *SAD* genes are involved in the desaturation of stearic acid (C18:0) to oleic acid (18:1) [19], *SAD (18830)* is likely the main contributor to oleic acid synthesis in sea buckthorn pulp. *SAD (26748)* also contributes to this process, especially in seeds.

### 2.5. FAD2 Expression

**Figure 3:**
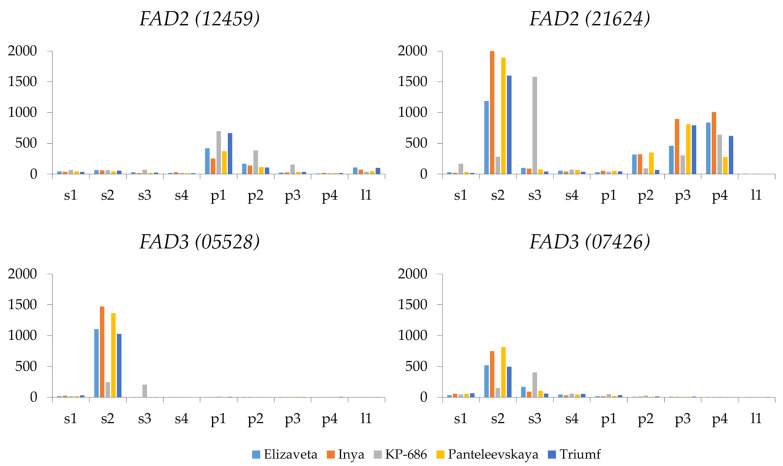
Expression of *FAD2 (12459)*, *FAD2 (21624)*, *FAD3 (05528)*, and *FAD3 (07426)* genes in seeds (s) and pulp (p) at four fruit ripening stages (1, 2, 3, 4) and in leaves (l) of five *Hippophae rhamnoides* varieties: Elizaveta, Inya, KP-686, Panteleevskaya, and Triumf. CPM (counts per million) values are shown on the y-axis.

Among the *FAD2* family, *FAD2 (21624)* exhibited the highest expression level in seeds and pulp, though it was not expressed in leaves (Figure 3 and Table S1). In pulp, *FAD2 (21624)* expression peaked at the third or fourth stage. In seeds, *FAD2 (21624)* expression peaked at the second stage (or the third stage for the variety KP-686). In addition, the expression level of this gene changed by more than 50-fold in seeds during fruit development. *FAD2 (12459*) exhibited increased expression at the first stage in pulp. However, its expression level remained low throughout subsequent stages in pulp and at all stages in seeds. *FAD2 (27005)* was barely expressed (Table S1). Since *FAD2* genes are involved in the desaturation of oleic acid (18:1) to linoleic acid (18:2) [20], *FAD2 (21624)* is likely the primary contributor to linoleic acid synthesis in seeds and pulp of sea buckthorn fruits.

### 2.6. FAD3 Expression

In the *FAD3* family, *FAD3 (05528)* and *FAD3 (07426)* genes were significantly expressed in seeds. Maximum expression levels were reached at the second stage of fruit development for most varieties and at the third stage for KP-686. These genes were practically not expressed in pulp (Figure 3 and Table S1). *FAD3 (10700)*, *FAD3 (24146)*, and *FAD3 (24879)* were practically not expressed in any of the studied organs or stages (Table S1). Since *FAD3* genes are involved in the desaturation of linoleic acid (18:2) to linolenic acid (18:3) [21], *FAD3 (05528)* and *FAD3 (07426)* are likely key genes in linolenic acid synthesis in sea buckthorn seeds.

### 2.7. FAD6 and FAD7/8 Expression

*FAD6 (25786)* and *FAD7/8 (02511)* genes were expressed at higher levels in pulp than in seeds. However, they were also significantly expressed in leaves. *FAD7/8 (02511)* was expressed about 10-fold higher in leaves than in fruits (Table S1). It is likely that the *FAD6* and *FAD7/8* genes do not play a significant role in the synthesis of linoleic and linolenic acids in sea buckthorn fruits.

### 2.8. FA Composition Analysis

**Figure 4:**
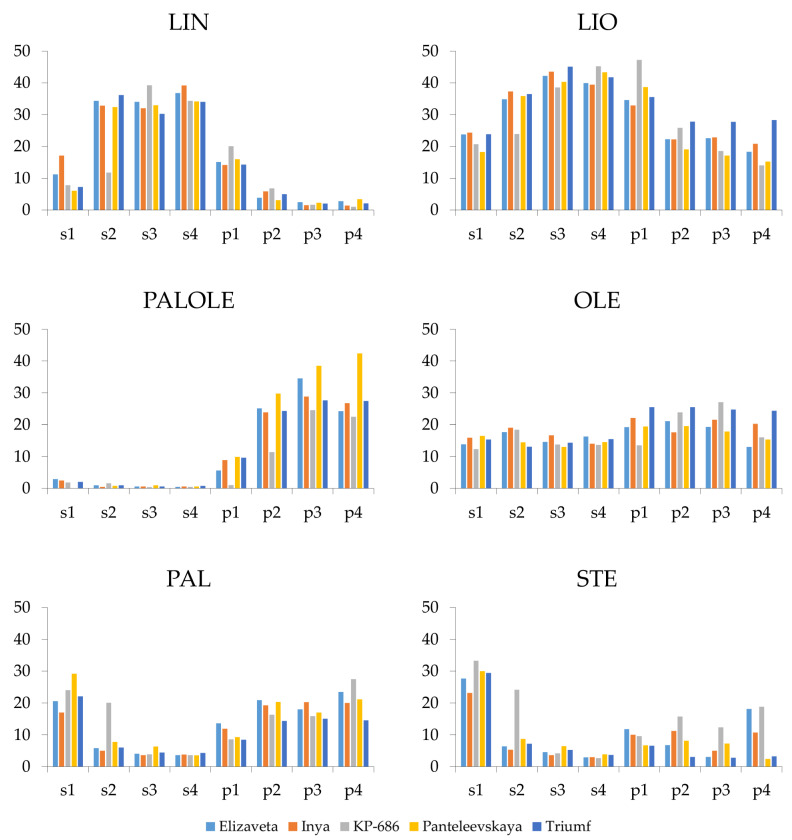
Content of the main fatty acids in seeds (s) and pulp (p) at four fruit ripening stages (1, 2, 3, and 4) of five *Hippophae rhamnoides* varieties: Elizaveta, Inya, KP-686, Panteleevskaya, and Triumf. Percentage values are shown on the y-axis. Average data for two biological replicates are shown. The fatty acids are: PAL—palmitic (16:0), PALOLE—palmitoleic (16:1), STE—stearic (C18:0), OLE—oleic (18:1), LIO—linoleic (18:2), and LIN—linolenic (18:3).

The FA composition of the oil was analyzed using the same samples of seeds and pulp of sea buckthorn fruits of varieties Elizaveta, Inya, KP-686, Panteleevskaya, and Triumf, for which transcriptomic analysis was performed. In general, common regularities were observed for all analyzed varieties: the FA composition of seeds differed significantly from that of pulp, and clear changes in the FA composition were observed during fruit ripening (Figure 4).

The content of linolenic acid (LIN) decreased in pulp during fruit development, reaching a minimum of 1–2% at the final two stages. In contrast, LIN content increased in seeds during ripening, reaching a maximum of 34–39% at the final two or three stages. In fact, mature sea buckthorn fruits are rich in LIN in seeds but not in pulp [1]. The obtained data on LIN content in sea buckthorn fruits aligns well with the data on the expression of *FAD3* family genes. In pulp, the levels of their expression was very low, but they were higher at the first stage of fruit development than at the other stages. Meanwhile, the maximum level of the most highly expressed *FAD3* gene—*FAD3 (05528)*—was reached in seeds of most varieties at the second stage of fruit ripening. This stage is probably key for LIN accumulation in seeds. FA composition data also confirm that *FAD3 (05528)* and, to a lesser extent, *FAD3 (07426)*, which were expressed at high levels in seeds but not in pulp, make a major contribution to LIN synthesis in sea buckthorn fruits.

Palmitoleic acid (PALOLE) exhibited different dynamic. In pulp, its content increased during fruit development and peaked at the final two or three stages. In seeds, PALOLE content was initially low and decreased to less than 1% during ripening. This is a natural occurrence since PALOLE is abundant in pulp but not in seeds of mature sea buckthorn fruits [1].

The linoleic acid (LIO) content in pulp decreased slightly during fruit development. It was the highest at the first stage but remained quite high at stages two-four. For most varieties, it was slightly less than 20%, but for Triumf, it was about 28%. Conversely, LIO content increased in seeds at later stages of ripening, reaching over 40%. The main increase in LIO content in seeds occurred at the second stage of fruit development when the *FAD2 (21624)* gene had the highest expression level.

Changes in oleic acid (OLE) content during sea buckthorn fruit ripening were not very pronounced in seeds and pulp. However, Triumf surpassed the other varieties in OLE content in pulp at all fruit development stages, with a content of about 24% for Triumf versus 14–20% for the other varieties. It should be noted that the expression level of the potentially key gene in OLE synthesis in pulp—*SAD (26748)*—was higher in Triumf than in the other studied varieties at later ripening stages.

In general, an increase in palmitic acid (PAL) content was observed in pulp during fruit development. For the variety KP-686, this increase was most pronounced, with the highest PAL level occurring at the fourth stage of ripening. In seeds, the content of PAL decreased from approximately 20% to 3–4% during fruit development.

The dynamics of changes in stearic acid (STE) content in seeds and pulp were generally similar to those in PAL content, with less accumulation in pulp.

It should be noted that the dynamics of FA accumulation in seeds and pulp differed for the variety KP-686 compared to the other studied varieties (including a shift to later stages of fruit development). This difference correlated with an altered gene expression pattern and was probably due to significant genetic differences between this variety and the other studied varieties [22].

### 2.9. Gene Expression and FA Composition

Thus, the *FAD3* genes, which are responsible for converting LIO to LIN, were barely expressed in pulp at any stage of development. *FAD3 (07426)* expression was slightly higher at the first stage (Table S1). The LIN content in pulp decreased during fruit ripening from an average of 16% at the first stage to an average of 2% at the fourth stage (Table S2). During ripening, sea buckthorn fruit size increases significantly (Figure S1), and the LIN content in pulp decreases due to the almost complete absence of *FAD3* expression. However, in seeds of most genotypes, LIN content increased several-fold from the first stage to the second, reaching an average of 34%, and changed slightly in later stages. The exception was KP-686, which had the highest LIN content at the third stage (about 39% vs. 12% at the second stage). *FAD3 (05528)* and *FAD3 (07426)* expression levels were the highest at the second stage in seeds of all analyzed genotypes except KP-686. KP-686 had the highest *FAD3 (07426)* expression level at the third stage and the maximum *FAD3 (05528)* expression level at the second and third stages. In other genotypes, *FAD3 (05528)* expression was low at the third stage. Therefore, the data on LIN content and the expression of *FAD3* genes were in concordance. The shift in LIN accumulation to a later stage in KP-686 was associated with a shift in the maximum expression levels of the *FAD3* genes. KP-686 is genetically distinct from the other studied sea buckthorn varieties and belongs to the Kyrgyz ecotype [22], which could explain its differences in *FAD3* expression profiles and LIN accumulation compared to other studied genotypes. However, more biological replicates are necessary to confirm these regularities for KP-686. Meanwhile, varieties Elizaveta, Inya, Panteleevskaya, and Triumf demonstrated very similar profiles of changes in LIN content and *FAD3* gene expression levels during fruit development. This suggests that the observed trends are reliable for these genotypes.

The LIO content changed similarly in the studied genotypes throughout fruit development. In seeds, LIO increased from the first to the third stage, after which it changed minimally. *FAD2 (21624)* gene, which is likely responsible for converting OLE to LIO in seeds, exhibited maximum expression at the second stage in all genotypes except KP-686, which exhibited maximum expression at the third stage, and then expression decreased significantly. It should be noted that seed size/mass decreased during sea buckthorn fruit ripening (Figure S1). Thus, the increase in LIO content in seeds at the third stage, when *FAD2 (21624)* expression was low, may be related to LIO concentration during seed ripening as well as to low *FAD3* gene (convert LIO to LIN) expression at the third stage. In pulp, LIO content was the highest at the first stage, decreased at the second stage, and changed slightly thereafter. Both *FAD2 (12459)* and *FAD2 (21624)* are probably involved in LIO synthesis in sea buckthorn pulp. *FAD2 (12459)* plays a more significant role at the initial stages, while *FAD2 (21624)* plays more significant role at the latter stages. The combined action of these genes resulted in a relatively constant LIO content during the second, third, and fourth stages of development. As with the *FAD3* genes, the expression of the *FAD2* genes shifted to a later stage in the variety KP-686. In addition, variety Triumf had the highest LIO content in pulp during the last three stages. However, the *FAD2* expression in Triumf did not differ significantly from that in the other studied varieties. This issue needs further investigation.

The OLE content was quite similar in seeds and pulp of sea buckthorn, though pulp had slightly higher levels. It changed very little during ripening. *SAD (26748)* was highly expressed in pulp, but not in seeds, and may be responsible for the higher level of OLE in pulp compared to seeds. The OLE content in pulp was higher in the variety Triumf than in the other studied sea buckthorn genotypes, especially at the fourth stage. Expression levels of *SAD (18830)* and *SAD (26748)*, which likely play a pivotal role in OLE synthesis in pulp, were also higher in Triumf than in the other varieties at the fourth stage. Thus, variety Triumf differed most from the other varieties in OLE content. Variety Triumf differs from Elizaveta, Inya, and Panteleevskaya in its selection history [22]. This may explain Triumf’s differences from these varieties. However, additional sets of fruit samples are necessary to confirm this observation.

PALOLE content was high in pulp but low in seeds. Of the studied genes, *SAD (26748)* had expression profiles quite similar to PALOLE content profiles during fruit development. *SAD (26748)* exhibited high expression levels in pulp but not in seeds. Based on these data, it could be suggested that *SAD (26748)* participates in the PALOLE synthesis. However, further research is necessary to clarify this.

Thus, the difference in FA composition between sea buckthorn seeds and pulp was likely due to the specific expression profiles of the studied *FAD3*, *FAD2*, *SAD*, and *FATA* genes. Genotype is probably also implicated in the FA composition of seeds and pulp, but additional studies are necessary to clarify this. The diverse expression levels of key FA synthesis genes in seeds and pulp may be due to transcription factors, which play an important regulatory role in oil synthesis [23,24,25].

The obtained in the present study results are important for a comprehensive analysis of tissue-specific gene expression in seeds and pulp of sea buckthorn fruits. Previous studies have identified important genes for FA synthesis in seeds or fruits of valuable crop plants, including olive [26,27,28], sunflower [29], sesame [30,31], cotton [32,33,34], oil palm [35,36]), walnut [37,38], yellowhorn [39], *Camellia oleifera* [40], flax [41], tree peony [42], and *Cyperus esculentus* [43,44]. These studies have greatly contributed to our understanding of the processes that determine the FA composition of oilseed crops and are essential for improving oil composition.

The biotechnology-based development of plants with modified oil FA compositions that better meet the requirements for healthy foods is a rapidly growing field [45,46,47]. Many studies have been conducted to create oilseeds with enhanced characteristics, including increased OLE content, using metabolic engineering techniques [48]. Our study obtained data on key genes involved in FA biosynthesis in sea buckthorn fruits, which can be used to develop improved varieties. Unlike many other plant species, increasing PALOLE (C16:1) rather than OLE (C18:1) in sea buckthorn pulp may be beneficial. Additionally, modeling the FA composition of the pulp to resemble that of the seeds, especially high LIN content, could be desirable. Furthermore, combining traditional breeding and marker-assisted selection can contribute to the development of sea buckthorn varieties with improved FA composition in their fruits.

## 3. Materials and Methods

### 3.1. Plant Material and RNA Extraction

The study examined sea buckthorn varieties that differed in fruit characteristics: Elizaveta, Inya, KP-686, Panteleevskaya, and Triumf (Figure S1). The fruits were collected at the Federal Altai Scientific Center of Agrobiotechnologies (Barnaul, Russia) at four stages of ripening: stage one on 30 June 2023, stage two on 14 July 2023, stage three on 2 August 2023, and stage four on 29 August 2023. Leaves were collected at stage one of fruit development. RNA was extracted from pools of at least ten samples of the same variety and stage using the CTAB method [49] with an additional step of washing the homogenized plant material with acetone.

### 3.2. cDNA Sequencing and Data Analysis

cDNA libraries were prepared for sequencing on the Illumina platform using the QIAseq Stranded RNA Lib Kit (Qiagen, Chatsworth, CA, USA). The quality and concentration of the cDNA libraries were assessed by a capillary gel electrophoresis using 2100 Bioanalyzer (Agilent Technologies, Santa Clara, CA, USA) and Qsep1-Plus (Bi-Optic, New Taipei City, Taiwan) and fluorometry using Qubit 4.0 (Thermo Fisher Scientific, Waltham, MA, USA). The cDNA libraries were considered suitable for sequencing if they exhibited a typical distribution of DNA fragment lengths and an absence of adapter dimers and had a concentration of at least 2 ng/μL. Sequencing was performed on NextSeq 2000 (Illumina, San Diego, CA, USA) with 50 + 50 nucleotide reads.

The analysis of the obtained reads was performed using the PPline [50] and RTrans (https://github.com/gskrasnov/RTrans, accessed on 1 May 2025) tools. The analysis included trimming the reads for quality and length, mapping the reads obtained for each sample to the annotated genome of *H. rhamnoides* (CNGB Nucleotide Sequence Archive, https://db.cngb.org/cnsa/, ID CNP0001846, accessed on 1 May 2025), and obtaining gene expression data in CPM (counts per million) format.

### 3.3. Fatty Acid Composition Analysis

The FA composition of oil in sea buckthorn fruit pulp and seeds was analyzed using a method adopted from Chagovets et al. [51]. Oil extraction was performed using methyl tert-butyl ether and methanol. Then, the lipid extract was hydrolyzed and an analysis on an ESI-Orbitrap mass spectrometer (Thermo Fisher Scientific) using the electrospray ionization method was performed. The mass spectra were processed using the Xcalibur 4.0 software (Thermo Fisher Scientific).

## 4. Conclusions

We evaluated the levels of primary FAs in seeds and pulp of five sea buckthorn varieties with different fruit characteristics. We also examined the expression levels of the *KAS*, *FAT*, *SAD*, *FAD2*, and *FAD3* gene families during fruit ripening. Significant differences were revealed between seeds and pulp for the studied traits. We identified genes with high expression levels that likely play a key role in FA synthesis in sea buckthorn seeds and pulp. Clear associations were revealed between the expression of particular genes from the studied families and the accumulation of FAs in sea buckthorn fruits. These results are important for understanding the reasons for the significant difference in FA composition between seeds and pulp and for developing sea buckthorn varieties with desirable FA composition using biotechnology.

## Data Availability

The raw sequencing data have been deposited in the NCBI Sequence Read Archive (SRA) under the BioProject accession number PRJNA1163394.

## Supplementary Materials

The following supporting information can be downloaded at: https://www.mdpi.com/article/10.3390/ijms262110396/s1.
