# Supplementary material for: Gene Expression and Fatty Acid Composition in Sea Buckthorn Seeds and Pulp During Fruit Development of Different Varieties
Source: Int J Mol Sci. 2025 Oct 26;26(21):10396. doi: 10.3390/ijms262110396 (PMC12607801; doi:10.3390/ijms262110396)
Supplement: Supplementary file 1 [file ijms-26-10396-s001.zip › ijms-3850870-supplementary/Figure S1 Fruits 2025.07.19.pdf]

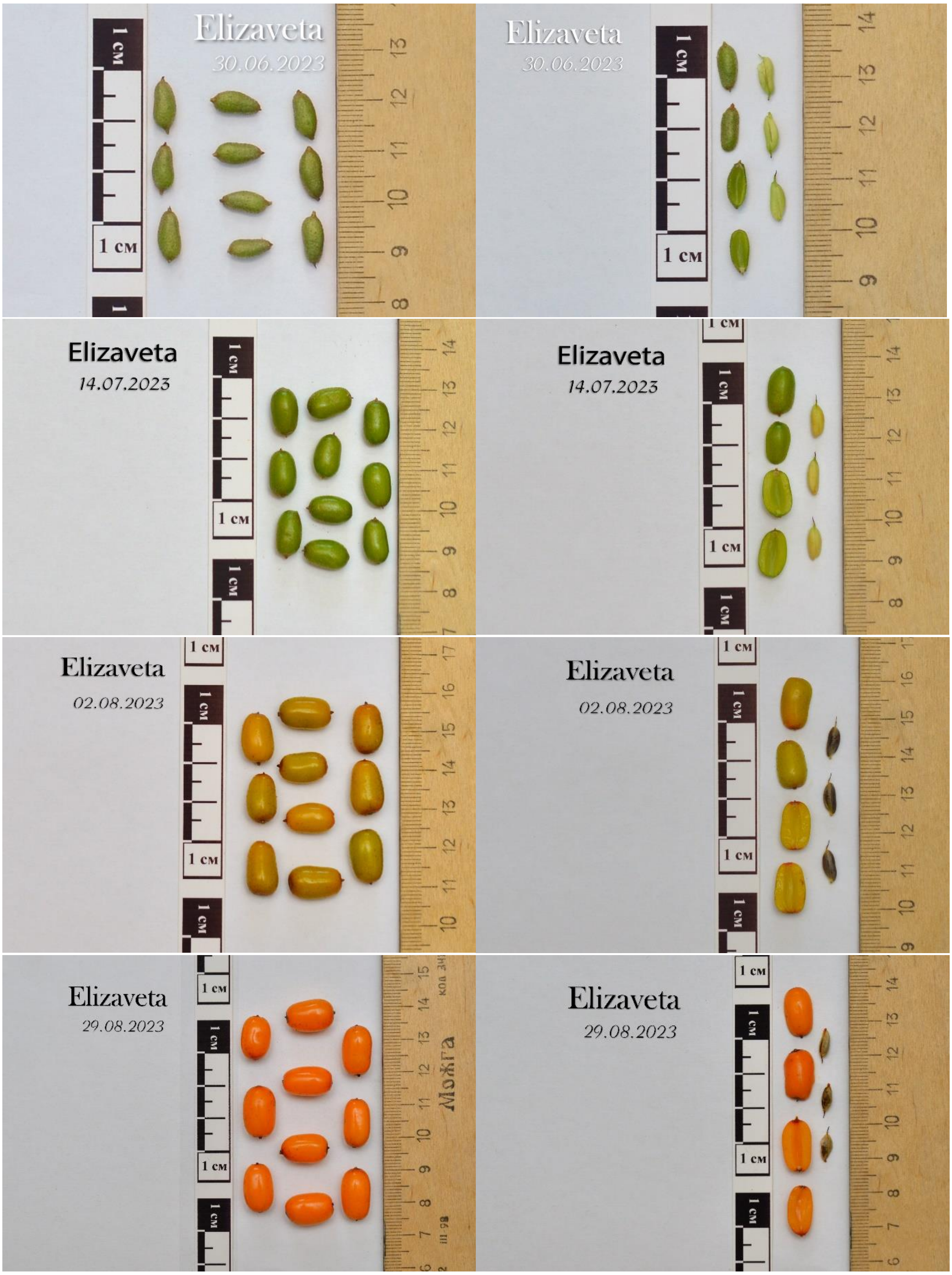

Figure S1a: Fruits, seeds, and pulp of sea buckthorn variety Elizaveta collected at the four main stages of fruit development.

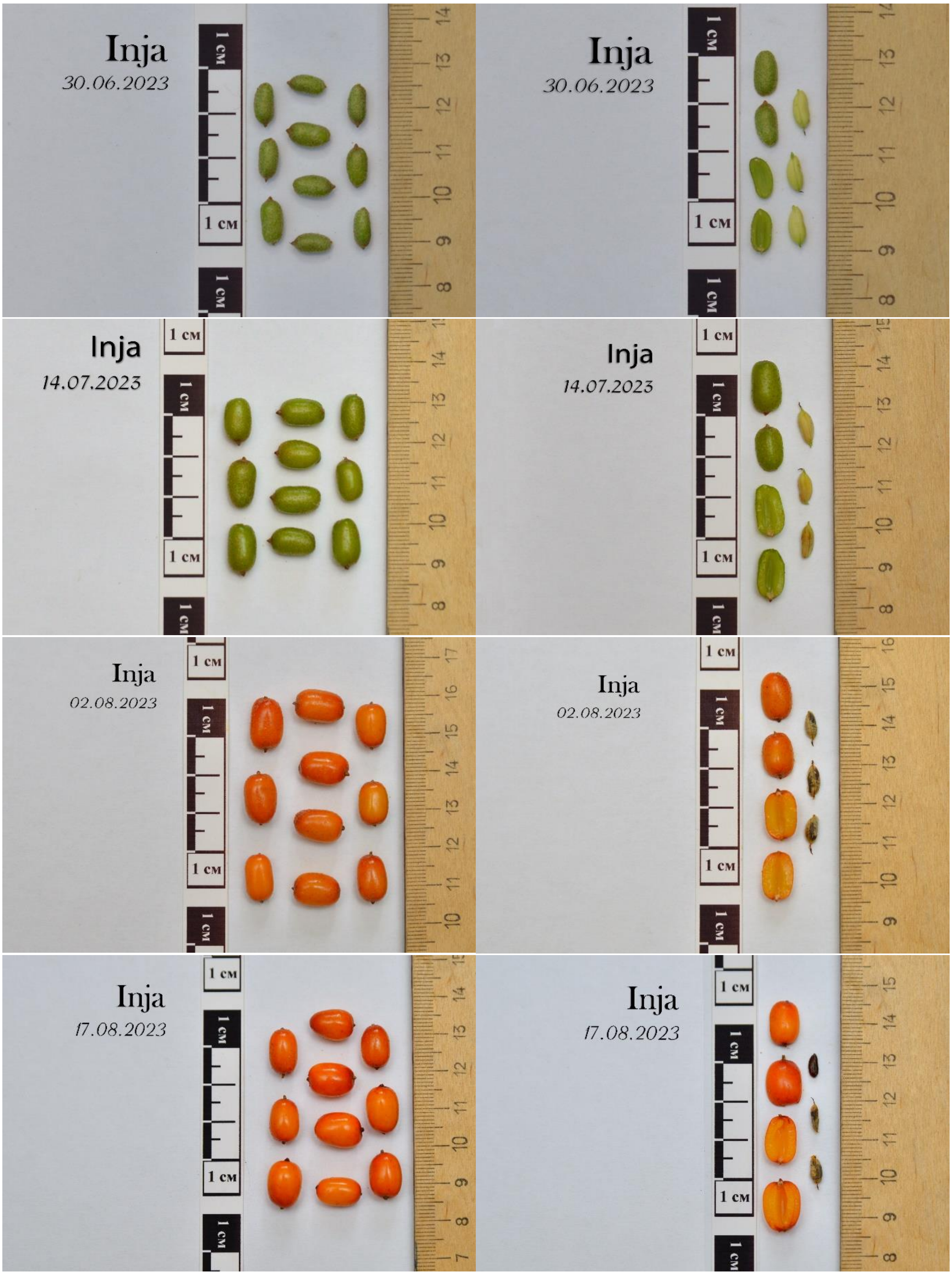

Figure S1b: Fruits, seeds, and pulp of sea buckthorn variety Inya collected at the four main stages of fruit development.

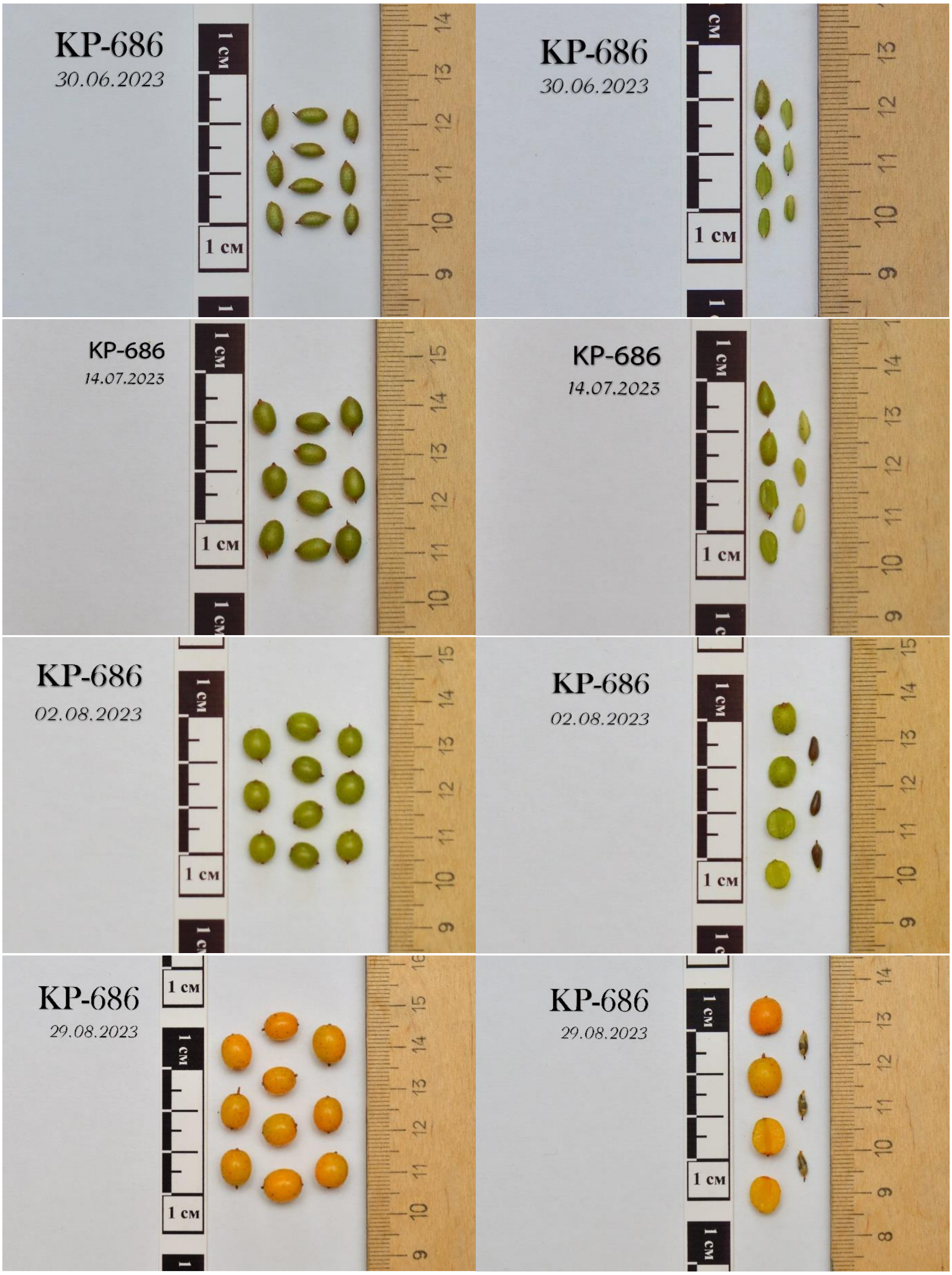

Figure S1c: Fruits, seeds, and pulp of sea buckthorn variety KP-686 collected at the four main stages of fruit development.

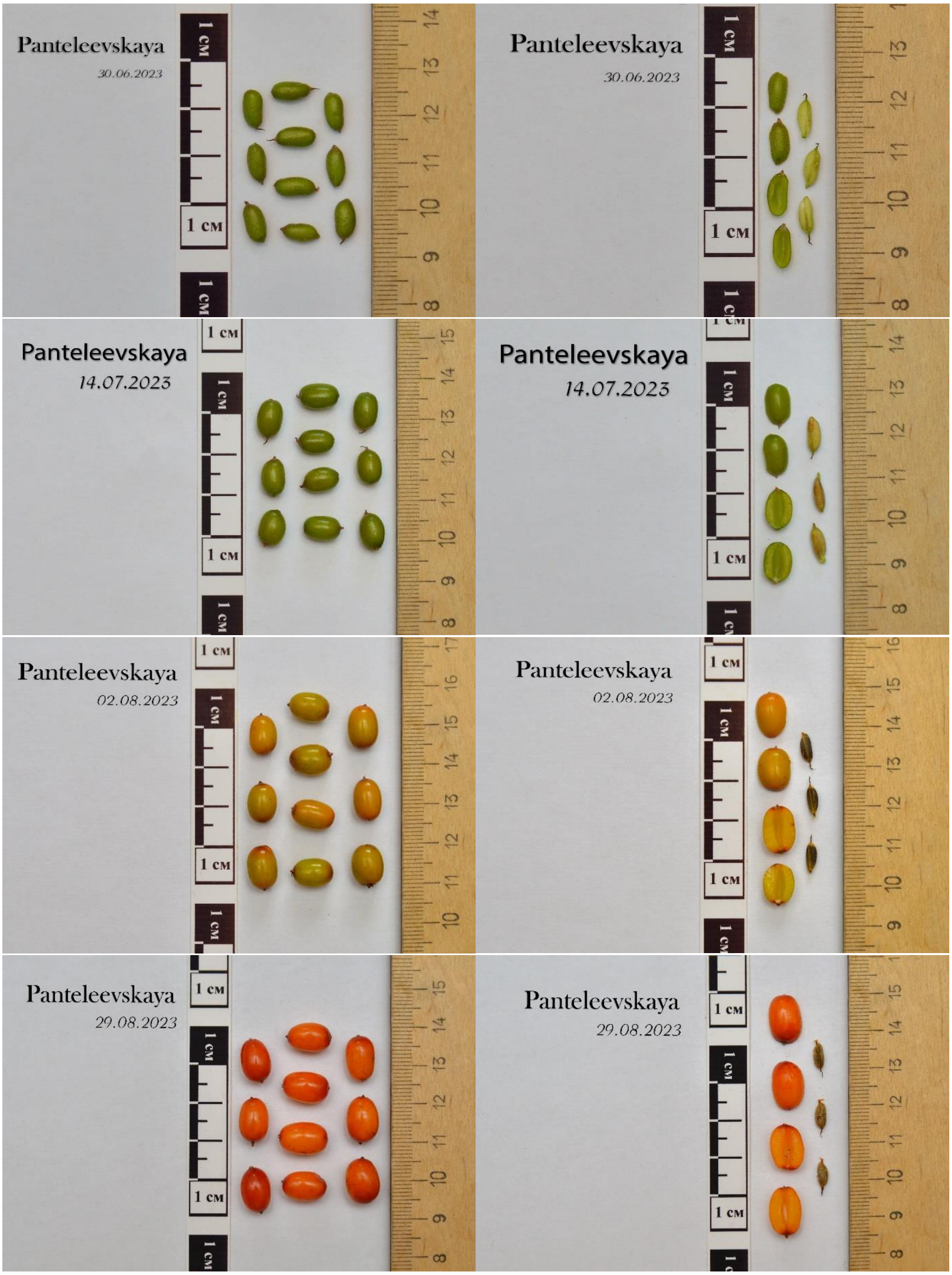

Figure S1d: Fruits, seeds, and pulp of sea buckthorn variety Panteleevskaya collected at the four main stages of fruit development.

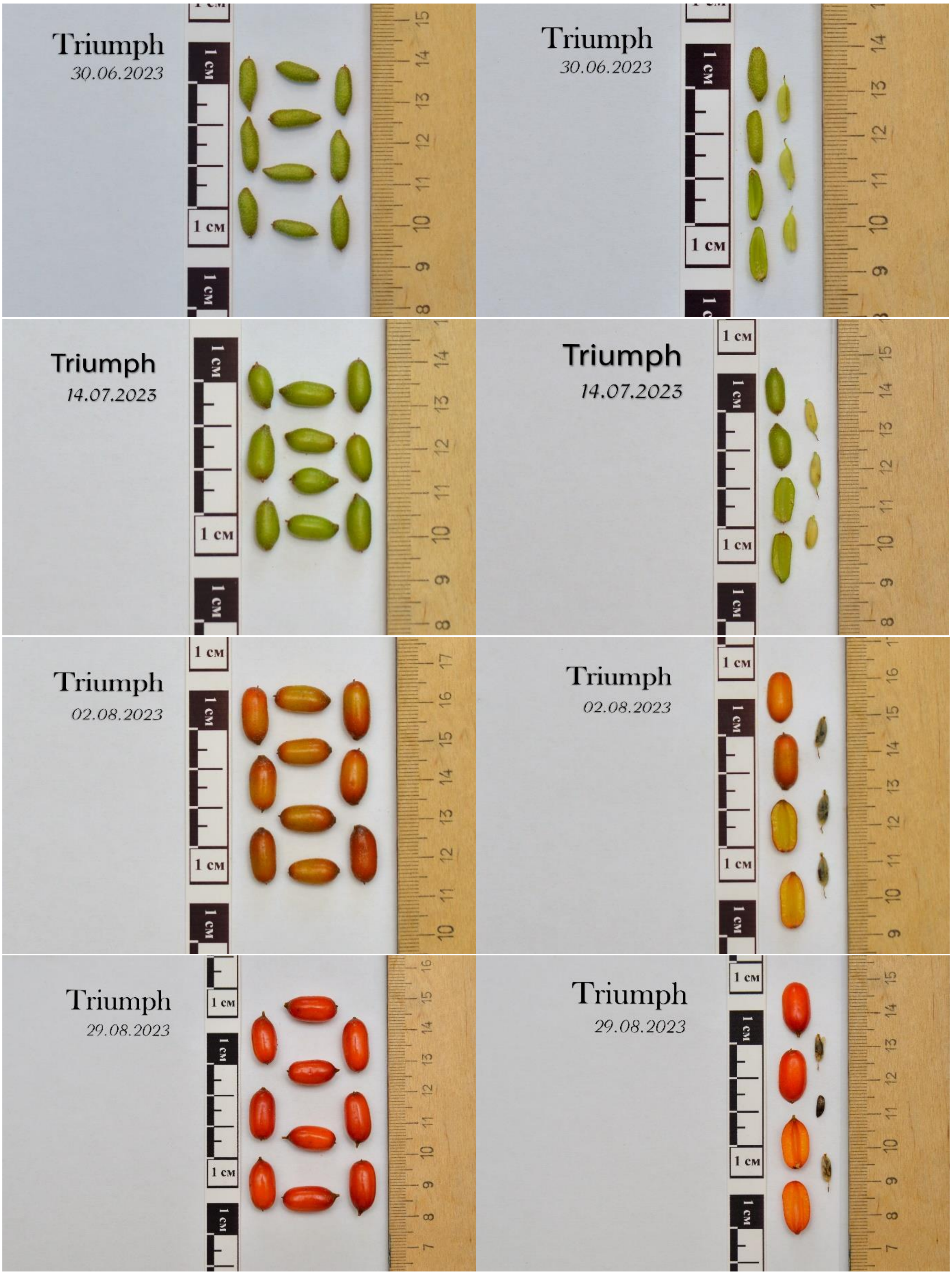

Figure S1e: Fruits, seeds, and pulp of sea buckthorn variety Triumf collected at the four main stages of fruit development.
